# In vitro somatic embryogenesis and plantlet regeneration from immature male inflorescence of adult *dura* and *tenera* palms of *Elaeis guineensis* (Jacq.)

**DOI:** 10.1186/s40064-015-1025-4

**Published:** 2015-06-12

**Authors:** Madhavan Jayanthi, Bollarapu Susanthi, Nandiganti Murali Mohan, Pranab Kumar Mandal

**Affiliations:** Indian Institute of Oil Palm Research, Indian Council of Agricultural Research (ICAR), West Godavari District, Pedavegi, 534550 Andhra Pradesh India; Indian Agricultural Research Institute, Indian Council of Agriucultural Research (ICAR), Pusa, New Delhi, 110012 India; National Research Centre on Plant Biotechnology, Pusa Campus, Indian Council of Agricultural Research (ICAR), New Delhi, 110012 India

**Keywords:** Oil palm, Propagation, Tissue culture, Somatic embryogenesis, Male inflorescence

## Abstract

We report here a method for plant regeneration through somatic embryogenesis from explants collected from immature male inflorescence of adult oil palm cultivated in India. Callus induction was successful from tissues of immature male inflorescence collected from both *dura* and *tenera* varieties of oil palm. A modified Y3 (Eeuwens) media supplemented with several additives and activated charcoal (3%) were used for the experiments. Out of four different auxin treatments, 4-amino-3,5,6-trichloro-2-pyridinecarboxylic acid (picloram) produced maximum callus induction (82%) and it was not significantly different from 2,4-dichlorophenoxyacetic acid (2,4-D) and a combination of 2,4-D + picloram. The callus induction obtained with auxin α-naphthalene acetic acid was only 54% and it was significantly low as compared to the other treatments. Highest embryogenesis was obtained with a combination of 2,4-D + picloram (4.9%) followed by picloram (3.4%). Genotypic variation in response to the same auxins was observed both for callus induction and embryogenesis. Callus induction and embryogenesis ranged from 42 to 72% and 6.8 to 9.35%, respectively in *tenera*. The formation of embryogenic calli was marked by the appearance of white to yellowish globular or nodular structures which subsequently formed clear somatic embryos. Somatic embryogenesis was asynchronous and at one time we could find different stages of embryogenesis like the globular, torpedo and the cotyledonary stages. The somatic embryos when exposed to light in the same basal media along with 6-benzyladenine (18 µM), abscisic acid (3.78 µM) and gibberellic acid (5.78 µM) regenerated into plantlets. To the best of our knowledge this is the first report o f callus induction and somatic embryogenesis from immature male inflorescence of oil palm.

## Background

Oil palm (*Elaies guineensis* Jacq) belonging to the family Arecaceae is the highest edible oil yielding crop (5–7 tons/ha) in the world. Its yield from a unit area could be up to 7–10 times more than that of any other annual oil seed crops. The palm oil is an edible oil with very unique properties like its trans-free nature, richest source of carotenoids and tocotrienols etc. Hence it is being used in several industries like food, bakery, soap, cosmetics, pharmaceuticals etc. Apart from that it is a promising resource for biofuel production (Basiron 2007). Due to its innumerable uses it has become the largest traded vegetable oil (Mielke 2013). Oil palm is a cross pollinated crop having a breeding cycle of more than 10 years and the propagation generally is by seeds. No method of vegetative propagation like cutting, layering, budding is possible in oil palm. Unlike date palm it also does not produce offshoots. The *tenera* palms, which are grown commercially in the farmers’ field is a hybrid derived by crossing *dura* X *pisifera* (DXP). Hence each palm is heterozygous in nature and genotypically different from each other (Low et al. 2008). To accelerate and capture the maximum yield potential of selected palms and to get uniform plants of elite palms, in vitro propagation needs to be followed.

The pathway of in vitro regeneration reported so far in oil palm is via somatic embryogenesis. There are a number of reports of in vitro regeneration from mature embryos (Teixeira et al. 1993, Thuzar et al. 2011, Balzon et al. 2013). However regeneration protocols from embryos cannot be used when clonal propagation is intended since we cannot predict the nature of the regenerants. Hence we need to use tissues from adult palms for tissue culture when clonal propagation is intended. Among the several explants which can be used for clonal propagation, inflorescence can be collected with minimal damage to the palm. Oil palm trees produce separate male and female inflorescences in leaf axils on the same palm in an alternating cycle of variable duration depending on genetic factors, age and environmental conditions. The female flowers eventually get pollinated and further become oil palm bunches. The male flowers of the palm on maturity produce pollen and eventually fall off. In vitro studies on oil palm inflorescence have been very limited and there is only one report of regeneration from female inflorescence of pisifera palms (Teixeira et al. 1994). In the present investigation we tested the effect of auxins on callus induction and embryogenesis from immature male inflorescence of *dura* and *tenera* palms. This is the first report of callus induction, somatic embryogenesis and plantlet regeneration from immature male inflorescence of oil palm.

## Methods

### Plant material and explants preparation

**Figure 1:**
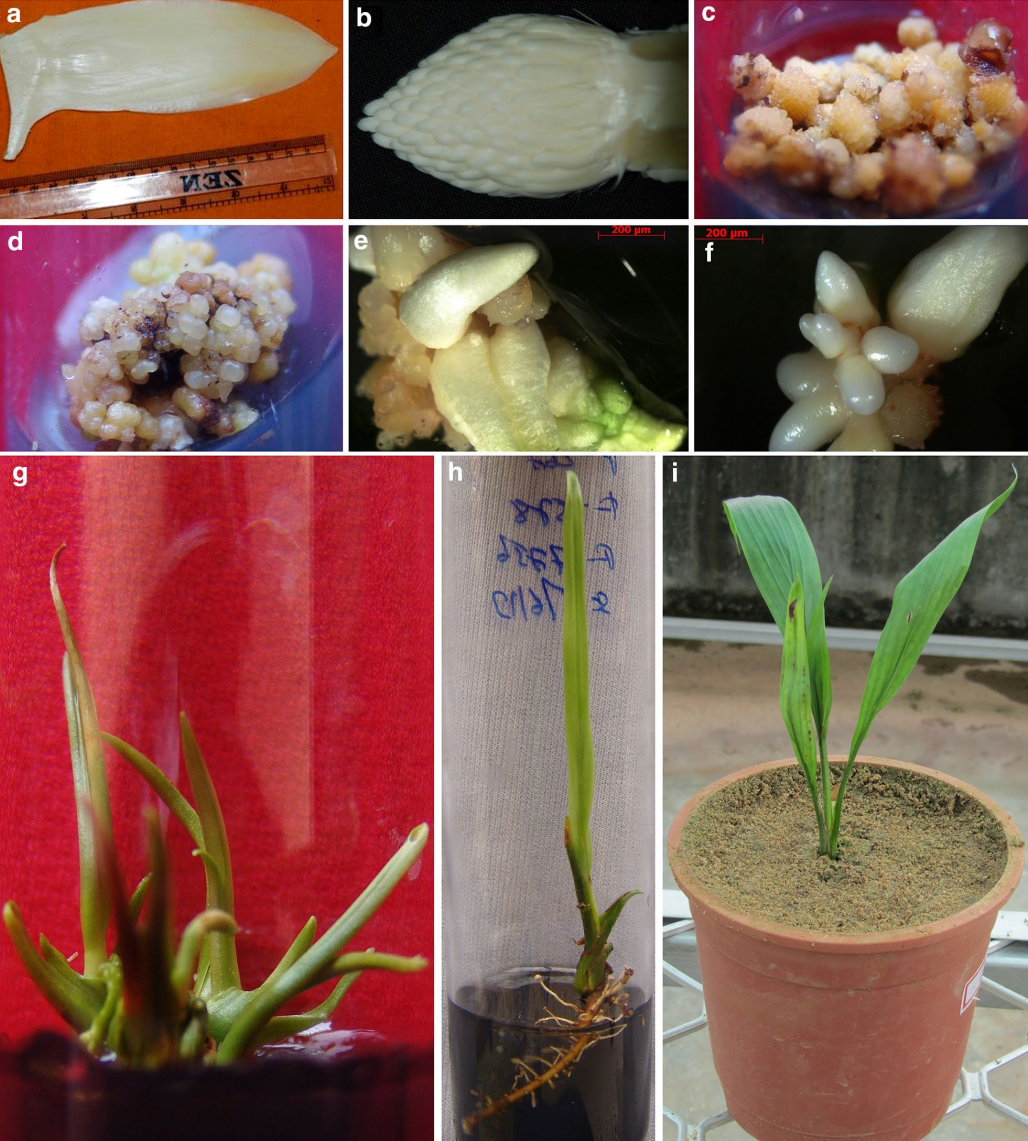
Somatic embryogenesis and plantlet regeneration from immature male inflorescence of oil palm (*Elaeis guineensis* Jacq). **a** Immature inflorescence collected from the leaf axil of *dura* palm. **b** Male inflorescence after removal of external and internal spathes. **c** Tissue swelling and initiation of callus on explants cultured in auxin supplemented media. **d** Embryogenic calli induction—note the development of globular calli. **e** Germination of somatic embryos. **f** Asynchronous somatic embryogenesis—note the heart and torpedo stages. **g** Plantlet regeneration from somatic embryos. **h** Rooting of regenerated plants. **i** Hardened plant of oil palm.

Three elite *dura* and two elite *tenera* palms (9 year old palms) as tall as 15–20 feet maintained in the experimental fields of this Institute were selected for this experiment. They were identified based on consistent high yielding nature (producing fresh fruit bunches >300 kg/year). The *dura* palms were designated as D1, D2 and D3 and the *tenera* palms as T1 and T2. The leaf number from the upper most exposed leaf was counted and the top most exposed leaf was considered as leaf number1. Three inflorescence (from the leaf axils between 8th and 15th leaf) of each palm was scooped out (Figure 1a) and transferred to a sterile polythene cover and brought to the laboratory. The external spathes were removed and the inflorescence still enclosed by the internal spathes, were surface-sterilized by immersion in 70% ethanol for 5 min, followed by air-drying in aseptic conditions. Thereafter, the internal spathes were removed and the male inflorescence alone were selected (Figure 1b) and cut into 1 mm pieces and used as explants. Three inflorescence were collected from each palm and explants from each inflorescence were considered as one replication. Explants were cultured in test tubes at the rate of 15 explants per tube. 50–100 tubes were used in each auxin treatment. Number of tubes varied due to variability in the size of the inflorescence.

### Plant culture media and culture conditions

The basal media used in these experiments was Y3 media (Eeuwens 1976) supplemented with the following additives (mg/l): myo-inositol (100), thiamine (0.1), pyridoxine(0.5), glycine (2), nicotinic acid (0.5), casein hydrolysate (500), l-glutamine (200), aspargine (200) arginine (200), sucrose (30,000) and activated charcoal (3000) along with auxins as mentioned below. For all media prepared the pH was adjusted to 5.8 with either 0.1 N NaOH or 0.1 N HCl prior to adding 0.8% (w/v) agar and they were sterilized at 121°C at 1.06 kg/cm^2^ for 15 min. The media was dispensed in test tubes and each test tube contained 20 ml media.

Four different auxin treatments such as 2,4-dichlorophenoxyacetic acid (2,4-D) (300 µM), 4-amino-3,5,6-trichloro-2-pyridinecarboxylic acid (picloram) (300 µM), a combination of 2,4-D + picloram (150 µM each) and α-naphthaleneacetic acid (NAA) (300 µM) was prepared for the experiments on callus induction. These treatments were designated as A1, A2, A3 and A4, respectively. In case of *tenera* palms, one auxin treatment (A3) was used for callus induction. The cultures were maintained in the dark at a temperature of 27 ± 1°C for a period of 16 weeks without subculture and the observations on callus induction were recorded as number of tubes with explants producing callus. Subculturing was carried out after every 4 months onto to the same basal media with the auxin concentration reduced to half on every subculture. The emergence of embryogenic calli and the different stages of embryos were observed and captured with the help of a Carl Zeiss Stemi Stereo microscope.

Embryogenic calli were transferred to regeneration media composing of the Y3 basal media along with 6-benzyladenine (BAP) (18 µM), abscisic acid (ABA) (3.78 µM) and gibberellic acid (GA_3_) (5.78 µM) and transferred to light conditions (4,000 lux provided by white fluorescent tubes of Philips) and maintained a 16 h light and 8 h darkness period at a temperature of 27 ± 1°C. Plants were regenerated from these embryogenic calli and after shoot growth of 4–6 cm were transferred to Y3 basal media with reduced concentration of activated charcoal (0.5 g/l) and increased concentration of sucrose (60 g/l) along with indole -3-acetic acid (IAA) (23 µM) and indole-3-butyric acid (IBA) (19.6 µM) for rooting. The well rooted plantlets having roots of 3–5 cm after sufficient growth were transferred to pots containing sterile agropeat and kept covered with a polythene sheet in the laboratory conditions for 1 or 2 months for primary hardening. Subsequently they were transferred to mist chamber where a temperature of 28 ± 1°C and relative humidity of 60% was maintained.

### Statistical analysis

In these experiments each individual palm is considered as a genotype. In the experiment with *dura* palms there were four auxin treatments with three replications (i.e. three different inflorescence from the same palm). Percentage callusing was calculated as percentage of tubes showing callus versus total number of tubes under each replication. Percentage embryogenesis was calculated as the percentage of tubes showing embryogenesis versus total tubes that showed callus. A factorial analysis with two factors, genotypes (different *dura* palms) and treatments (auxins) was done. Critical difference or CD was calculated for the four auxin treatments. Similarly we also calculated the critical difference (LSD) of genotype X auxin interaction and calculated the range for all 12 means. Means were compared using least significant difference test (LSD: p < 0.05). In case of *tenera* there were only two palms and three inflorescence from each palm were used and hence a *t* test was performed to compare the means of callus induction and embryogenesis obtained from two palms.

## Results and discussion

**Figure 2:**
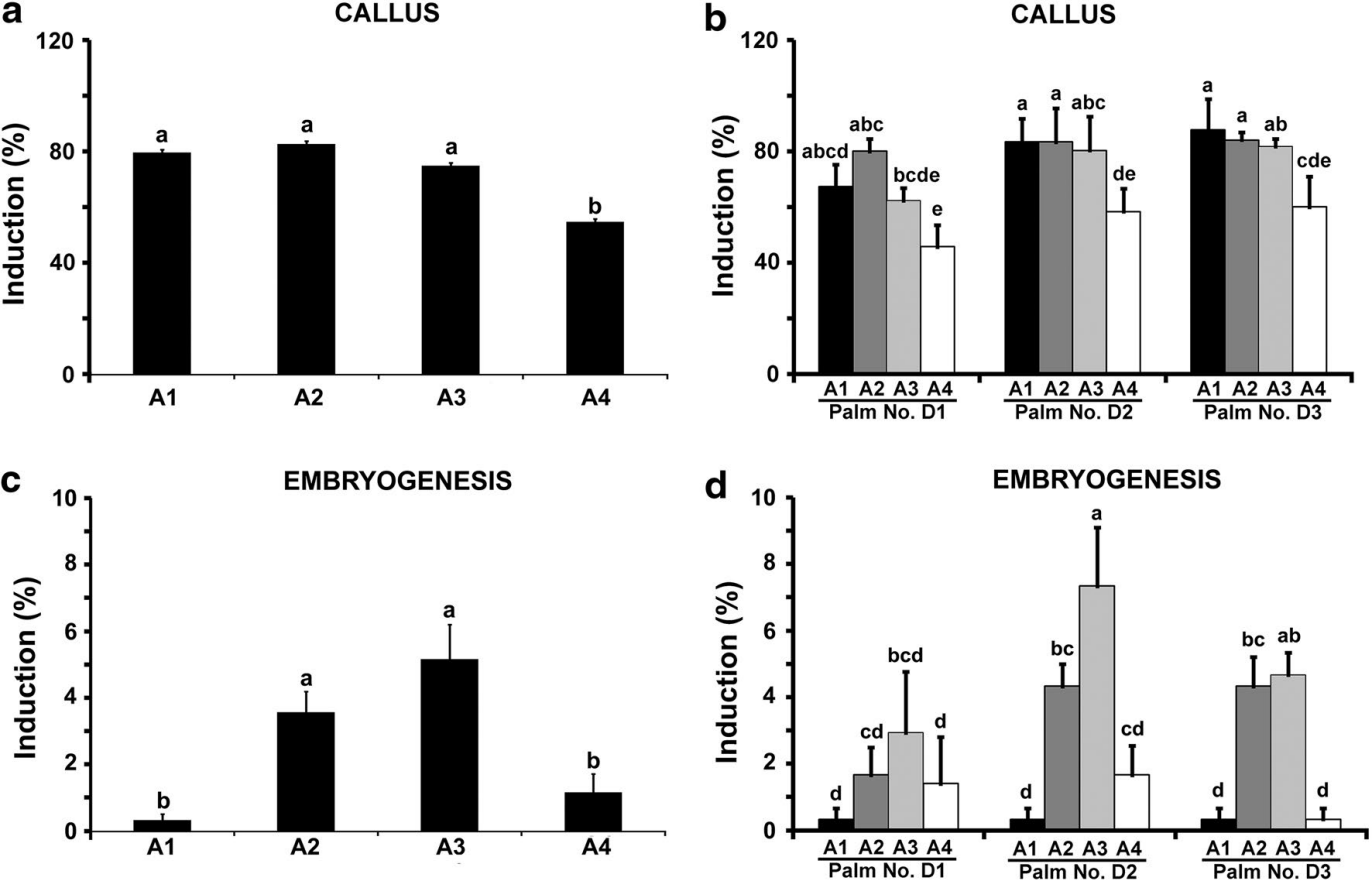
Effect of different auxins on callus induction and embryogenesis from immature male inflorescence of *dura* palms. *A1*, *A2*, *A3* and *A4* represents 2,4-D, picloram, 2,4-D + picloram and NAA, respectively. *D1*, *D2* and *D3* represents the three *dura* palms selected for the study. Means were compared using least significant difference test (LSD: p < 0.05). Means with the same letter are not significantly different from each other. **a** Effect of different auxins on callus induction. **b** Analysis of callus induction obtained with different auxins in *dura* palms. **c** Effect of different auxin treatments on embryogenesis in *dura* palms. **d** Analysis of embryogenesis percentage obtained with different auxins in *dura* palms.

The regeneration protocol reported here via indirect somatic embryogenesis has three major developmental stages: induction of callus, induction of embryogenic callus, somatic embryo maturation, and plantlet regeneration. Auxins are critical for the callus induction from explants of oil palm which is the first step in the oil palm tissue culture process. There was tissue swelling and profuse growth within 3–4 months and callus induction was observed from the cut edges (Figure 1c) in all the four different auxin treatments viz., A1, A2, A3 and A4. The overall callus induction percentage ranged from 54.67 to 82.44 percent in *dura* palms (Figure 2a). Callus induction obtained with 2,4-D was not significantly different from that obtained with picloram and the combination of 2,4-D + picloram, which indicated that the effect of picloram as auxin was similar to 2,4-D in inducing callus. The auxin NAA also could induce callus but it was significantly less as compared to other auxins (Figure 2a). There were genotypic differences in responses to auxins. Data analysis of callus induction is given in Figure 2b. In D1 palm the callus induction ranged from 45.67 to 80%. In this palm the best callus induction was obtained with picloram. In D2 palm both 2,4-D and picloram are able to induce the maximum callus induction of 83.3% and were on par to each other. In D3 palm the best callus induction was 86.67% obtained in media with 2,4-D and it was not significantly different from that obtained with picloram (84%). Callus induction with NAA was significantly lower in all the palms. In case of two *tenera* palms, the callus induction obtained was ranging from 42 to 72% on Y3 media with both 2,4-D and picloram (Table 1). The callus induction obtained with T1 tenera palm was better than T2 palm on the same media and was significantly different. Teixeira et al. (1994) reported a callus induction percentage ranging from 6 to 50 from immature female inflorescence of oil palm when the 2,4-D concentration ranged from 400 to 500 µM in MS (Murashige and Skoog 1962) media. The callus induction obtained in our experiments using Y3 media was much higher than reported earlier. Activated charcoal is the main antioxidant that is used for absorption of phenolic compounds in palm tissue culture and its use has been reported in all palms (Valverde et al. 1987; Karun et al. 2004; Huong et al. 1999; Steinmacher et al. 2007; Luis and Pereira 2014). However the use of activated charcoal should be coupled with the use of high concentration of auxins since most of these auxins are also absorbed by activated charcoal. Thuzar et al. (2012) reported that use of 100–140 mg/l of 2,4-D in the presence of activated charcoal will effectively supply only 2–2.8 mg/l of the auxin and the remaining will be absorbed by activated charcoal. In our experiments we found that activated charcoal at 0.3% was effective in controlling oxidation and thereby improving the callus induction percentage. These calli had to be subcultured after every 4 months to a fresh media with reduced concentration of auxins. After every subculture it was found that there was an increase in oxidation of calli and later the tissues again started responding. The emergence of embryogenic calli was observed after fourth subculture and continued until sixth subculture. The observations on the percentages of cultures showing embryogenesis were taken at the end of sixth sub culture. The embryogenic calli induction was characterised by the appearance of white to yellowish globular or nodular structures with suspensor region (Figure 1d). By the end of fourth subculture, these embryogenic calli began to multiply and proliferate rapidly (Figure 1e). Asynchronous development of somatic embryos was observed when observed under the microscope. Different stages of somatic embryos including the heart shaped and torpedo shaped embryos were clearly visible (Figure 1f). The data obtained on embryogenic calli was analyzed and the data revealed that 2,4-D + picloram induced the highest embryogenesis of 4.9% followed by picloram at 3.4%. Both 2,4-D and NAA induced low embryogenesis and were not significantly different from each other (Figure 2c). The overall embryogenesis percentage ranged from 0.33 to 4.98 in *dura* palms. Genotypic analysis among *dura* palms revealed that D2 palm was more potent for embryogenesis (7.3%) compared to other two palms (Figure 2d). In all the palms it was found that the combination of 2,4,-D and picloram could induce the maximum embryogenesis. Though the auxins 2,4,-D and picloram individually could induce maximum callus induction in *dura* palms, maximum embryogenesis was obtained only with the combination of 2,4-D + picloram in all the three palms. Hence, this was selected as most potent treatment and used for *tenera* palms. In case of *tenera* palms, the embryogenesis ranged from 6.8 to 9.35% with 2,4-D + picloram in Y3 (Table 1). The embryogenesis percentage obtained with *tenera* palms was better than *dura* palms on the same media. Among the *tenera *palms T1 palm showed a better callus induction and embryogenesis percentage.

**Table 1:** Callus induction and embryogenesis percentages in tenera palms

	T1	T2
Callus induction (%)	71.74 (3.59)*	41.7 (1.84)*
Embryogenesis (%)	9.35 (0.78)	6.8 (1.90)

The values are means of three replications (i.e. three inflorescence from the same palm). The figures in parenthesis are standard error means and * indicates that they are significantly different. The number of tubes used in each replication was 50–100.

Oil palm tissue culture is still a challenging process since it is found that certain genotypes do not respond to culture conditions and are not embryogenic (Thuzar et al. 2012). Also this tissue culture process is affected by numerous culture conditions, including culture medium composition, genotype, type and concentration of growth regulators etc. (Feher et al. 2003). In our study it was found that our genotypes were showing a high percentage of primary callus induction which has not been so far reported and also they were embryogenic. Teixeira et al. (1994) reported that while primary callus induction from immature inflorescence was possible in MS media, embryogenic calli induction was possible only in Y3 media. The superior effect of picloram in combination with activated charcoal for induction of embryogenic callus induction has been reported from many monocots and palms (Karun et al. 2004; Huong et al. 1999; Steinmacher et al. 2007; Luis and Pereira 2014; Beyl and Sharma 1983; Fitch and Moore 1990; Groll et al. 2001). Superiority of this auxin over the other auxins has been attributed to the effective uptake and mobilisation of this growth regulator and rapid mobilisation at target sites (Karun et al. 2004).

On transfer to light in regeneration media the well developed somatic embryos regenerated into plantlets (Figure 1g). After sufficient shoot growth, they were transferred to Y3 basal media with reduced concentration of activated charcoal (0.5 g/l) and increased concentration of sucrose (60 g/l) along with IAA (23 µM) and IBA (19.6 µM) for rooting. The rooted plantlets (Figure 1h) of ≥10 cm shoot and >5 cm root were transferred to sterile soil in pots covered with polythene bag and kept in the laboratory conditions for a month. The established plants were transferred to pots containing sterile soil and transferred to mist chamber (Figure 1i).

Male inflorescence was chosen since it was found in our preliminary work there was lesser oxidation in male inflorescence as compared to female inflorescence and also better response for callusing. These were observed in several repeated experiments. Male inflorescence apart from producing pollen is not of much use to the palm since it is the female inflorescence that forms the fruits and bunches. Hence collection of male inflorescence will not be a loss to the palm and also it can be collected with minimal damage to the palms.

## Conclusion

The significant finding in this research is as follows: (1) Use of an explant which can be collected with minimal damage to palm. (2) We could obtain a high percentage of primary callus induction from immature male inflorescence. (3) The use of picloram for callus and embryogenesis from inflorescence was found to be advantageous. The strategy for mass multiplication will be to reclone these initial plantlets after sufficient growth, so that enough explants can be obtained from a specific palm (genotype). The protocol reported here opens up the prospects of using these easily available explants for large scale multiplication of oil palm.

## Abbreviations

Picloram: 4-amino-3,5,6-trichloro-2-pyridinecarboxylic acid
2,4-D: 2,4-dichlorophenoxyacetic acid
NAA: α-naphthaleneacetic acid
BAP: 6-benzyladenine
IBA: indole-3-butyric acid
IAA: indole -3-acetic acid
ABA: abscisic acid
GA_3_: gibberellic acid
